# Paraneoplastic Polyarthritis in a Patient with Synchronous Lung and Colorectal Malignancy

**DOI:** 10.31138/mjr.20230831.pp

**Published:** 2023-08-31

**Authors:** Claudia Cobilinschi, Alexandra Constantinescu, Egor Șargarovschi, Simona Enache, Andra Rodica Bălănescu, Daniela Opriş-Belinski

**Affiliations:** 1 Department of Internal Medicine and Rheumatology, Sf. Maria Clinical Hospital, Bucharest, Romania,; 2 Carol Davila University of Medicine and Pharmacy, Bucharest, Romania,; 3 Department of Thoracic Surgery, Sf. Maria Clinical Hospital, Bucharest, Romania,; 4 Department of Histopathology, Sf. Maria Clinical Hospital, Bucharest, Romania

**Keywords:** paraneoplastic polyarthritis, synchronous tumours, hypertrophic osteoarthropathy, Caplan’s

## Abstract

Arthritis is an unusual manifestation of paraneoplastic syndrome, appearing in a variety of cancers, including pulmonary and colorectal. It can often pose a diagnostic challenge to physicians, since it may be difficult to distinguish from more commonly encountered rheumatic illnesses. Moreover, synchronous cancers are rare and unexpected in patients with symmetrical polyarthritis. Hypertrophic pulmonary osteoarthropathy is to be considered in patients with polyarthritis and lung neoplasia. The aim of this report is to highlight the case of a patient presenting with paraneoplastic polyarthritis, which led to identifying the presence of underlying synchronous lung and colorectal malignancies. Lymph node biopsy was performed raising suspicion of Caplan’s syndrome but lung lobectomy confirmed adenocarcinoma. Rheumatologists should be reacquainted with rheumatic manifestations in malignant diseases.

## INTRODUCTION

Paraneoplastic syndromes occur as the result of tissue damage at distant sites from the primary tumour. They can often precede cancer diagnosis, though they may also occur at the same time, develop after the diagnosis has been made or be an early sign of recurrence. A variety of paraneoplastic manifestations affecting the joints could mimic primary rheumatic diseases, making differential diagnosis difficult, yet essential in the early detection of underlying malignancy.^1,2^

While arthritis is one of the most frequent complaints encountered by rheumatologists, paraneoplastic arthritis is not a common occurrence. It is usually seronegative, predominantly appearing in males and the average age of onset is around the fifth decade. Asymmetrical oligoarthritis is the usual clinical presentation, particularly involving the lower limb joints, though cases of symmetric polyarthritis mimicking rheumatoid arthritis have also been reported. Response to conventional DMARDs, corticosteroids or non-steroidal anti-inflammatory drugs is typically poor, but symptoms usually improve with treatment of the underlying condition.^1,3^

Synchronous cancers are primary malignancies that occur within a short timeframe of each other, typically under six months, with the colon being the most commonly affected organ.^4,5^

Cigarette smoking is a known risk factor for a variety of cancers, especially the lung cancer and rheumatic diseases including rheumatoid arthritis.^6,7^

## CASE REPORT

A 60-year-old male patient presents with symmetric polyarthritis affecting the small joints of the hands and ankles getting progressively worse, fatigue, and significant weight loss of approximately 20 kg in the last three months. The patient confirms being a former long-time smoker, estimated at 45 pack-years.

Prior to admission in our department, the patient was evaluated for similar symptoms and laboratory testing revealed mild normochromic, normocytic anaemia, and high inflammatory markers, while the rheumatoid factor and ACPA antibodies were within normal range. He was diagnosed with seronegative rheumatoid arthritis and started on immunosuppressive treatment with leflunomide and oral corticosteroids, with no significant improvement of joint manifestations. Leflunomide was eventually stopped due to intense nausea.

**Figure 1.**
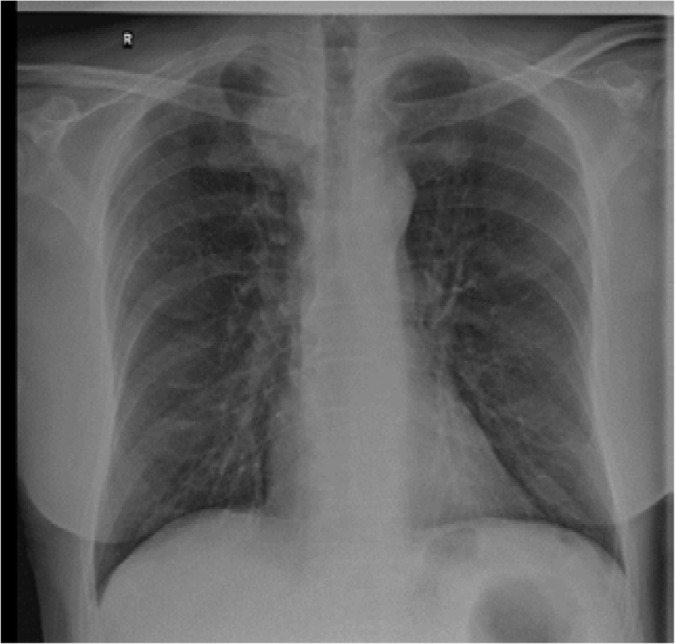
Chest X-ray showing round mass in upper right lobe.

A chest x-ray revealed a round radiopaque mass in the upper right lung, which was not further investigated (**Figure 1**).

**Figure 2.**
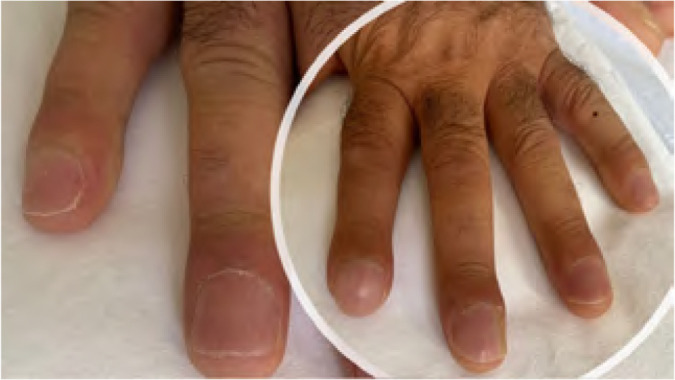
Clubbing of the fingers.

Upon admission to our hospital, digital clubbing was observed during clinical examination (**Figure 2**). Laboratory findings were consistent with prior testing, with persistently high inflammatory markers. Hand x-ray revealed osteoarthritic changes but no erosions suggestive of rheumatoid arthritis. Ultrasound imaging revealed active synovitis of the anterior recesses of the ankles and the first metatarsophalangeal joints.

A pneumology consult with functional respiratory tests confirmed distal pulmonary obstruction syndrome, while echocardiography identified a small amount of pericardial effusion.

**Figure 3.**
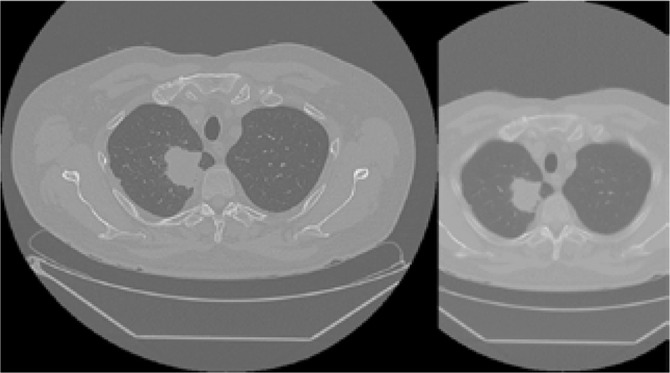
Thoracic CT scan showing a tumoral mass in the upper lobe of the right lung.

Multiple colonic polyps were identified with lower endoscopy, ultimately revealing in situ colorectal carcinoma. Whole body CT scan identified a large tumoral mass suggestive of malignancy in the right superior lobe, alongside multiple mediastinal enlarged lymph nodes (**Figure 3**).

The patient was further referred to thoracic surgery. Bronchoscopy and cervical mediastinoscopy procedures were performed during the same anaesthesia, for endobronchial and mediastinal pathologic staging of the primary lung tumour. Mediastinal paratracheal ganglia were biopsied, with pathology testing identifying anthracotic pigment compatible with the patient’s long-time smoking history.

**Figure 4.**
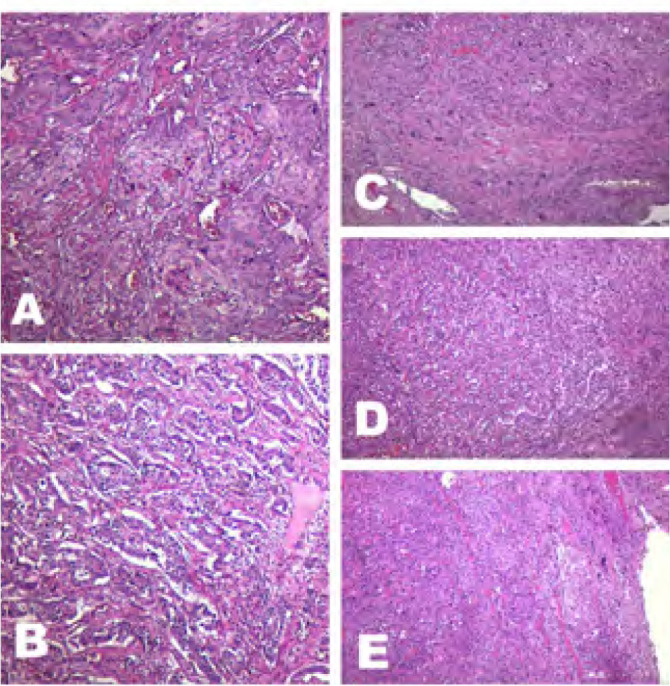
Hematoxylin and eosin staining of adenosquamous carcinoma with 20x magnification: **(A)** Keratinising squamous carcinoma component; **(B)** Adenocarcinoma component; **(C)** Pleomorphic carcinoma component; **(D & E)** Solid adenocarcinoma component.

After full recovery from first surgical approach, the patient underwent a radical right upper lobectomy with systematic mediastinal lymphadenectomy. Pathology testing results of the resected tumour and excised lymph nodes revealed a mixed type of lung carcinoma: adenosquamous carcinoma of the lung with invasion of the visceral pleura, associating components of moderately differentiated focal keratinising squamous carcinoma (approximately 60% of the tumoral mass), adenocarcinoma with an acinar and solid pattern, as well as small areas of pleomorphic carcinoma TNM pT3N2M0, stage IIIA (**Figure 4**).

The patient had a favourable post-operative recovery, was referred to an oncologist, and is currently due to start adjuvant chemotherapy.

As a result, the nature of the arthritis was deemed to be paraneoplastic. The possibility of Caplan’s syndrome was initially considered given the symmetrical polyarthritis and lung mass with anthracotic pigment in lymph nodes. Yet it was ultimately excluded, as the patient did not have a history of exposure to toxic agents, such as asbestos, silica, or coal, while the diagnosis of rheumatoid arthritis was ruled out based on biochemical and imaging findings.

## DISCUSSION

Arthritis is one of the most common presenting symptoms in medical departments. Paraneoplastic arthritis (PA) is part of a heterogenous group of rheumatic paraneoplastic syndromes, which are not a result of direct tumoral or metastatic invasion. These disorders are often difficult to distinguish from typical rheumatic diseases, sometimes preceding the diagnosis of underlying cancer by months or even years; sometimes they can be the first sign of recurring malignancy. Usually, cancer will manifest itself within approximately three months after joint symptoms. Timely recognition of paraneoplastic processes can be key in detecting early-stage cancers that are more easily treatable. A high index of suspicion is crucial in middle-aged patients, particularly smokers, presenting with arthritis.^8,9,10,11^

Several hypotheses have been considered in determining the mechanism of paraneoplastic syndromes but it seems they are either the result of tumoral secretion of humoral mediators (hormones or peptides), or the consequence of antibody cross-reactivity between targeted tumoral cells and regular host cells^12^.

PA has been associated with several neoplasms, including gastric, colon, pancreatic, ovarian, breast and laryngeal, but it is most frequently identified in patients with lung cancer. As of yet, no correlation has been observed between the size of the tumour and the severity of paraneoplastic manifestations. Some studies have claimed that PA is more frequently observed in men, age of onset can range from 43 to 76 years and typically correlates with that of the associated malignancy.^8,13^

In contrast, onset of rheumatoid arthritis (RA) usually occurs between 30 and 55 years of age, with women being more frequently affected. However, both RA and PA present with morning stiffness, joint swelling and impaired function of the affected joints. High inflammatory markers and anaemia are common findings in both disorders. Elevated rheumatoid factor may also be present in patients with PA, since it occurs in 10 to 20% of patients with malignancy, further complicating differential diagnosis.

Hypertrophic osteoarthropathy (HOA) is a rare occurrence, defined by the triad of digital clubbing, periostitis and arthropathy.^12^ Primary HOA is a genetic disorder with either autosomal dominant or recessive inheritance and accounts for approximately 5% of all HOA cases. Secondary HOA usually develops in the context of malignancy or cardiovascular disease.^13^ When observed in the context of lung disease, it is called hypertrophic pulmonary osteoarthropathy (HPO). Pulmonary malignancy accounts for 70–80% of HPO cases, although chronic respiratory disease, cystic fibrosis, and congenital cyanotic heart disease can also be responsible. The most promising theory explaining the mechanism of HPO involves megakaryocyte fragments that bypass the pulmonary capillary network and enter systemic circulation, ultimately interacting with endothelial cells, stimulating the release of growth factors, such as platelet-derived growth factor (PDGF), vascular endothelial growth factor (VEGF) and prostaglandin E, leading to fibroblast proliferation, new bone formation and digital clubbing. Notably, HPO is differentiated from RA by the lack of joint erosions on radiographic imaging and a negative rheumatoid factor. HPO is also usually unresponsive to conventional analgesics, non-steroidals, or corticosteroids. The best solution is tumoral resection, although bisphosphonates, particularly pamidronate, have been reported to be efficient in alleviating pain and swelling in patients with HPO.^2,12^

Synchronous cancers are primary malignant tumours that develop within less than six months interval from each other, the colon being the most affected.^4^ Approximately 3.5% of patients with colorectal carcinoma will develop two primary colorectal tumours.^5^ However, synchronous malignancies can sometimes occur in different sites, such as both lung and colon neoplasms.^14,15^ One study found that colon cancer was identified within one month in 0.54% of patients diagnosed with pulmonary malignancy.^14^

Smoking is associated with an increased risk of neoplasia in most organs, including the lungs and colon.^6,16^ In patients presenting with arthritis, maintaining a high index of suspicion, specifically in those with a history of smoking, could lead to an early diagnosis of underlying malignancy. The importance of a plain chest radiography should therefore not be underestimated in such cases.^6,17,18^

Paraneoplastic polyarthritis can be a diagnostic challenge for rheumatologists, particularly as it is an unusual occurrence, and it may closely mimic other rheumatic diseases. It should be taken into account in any patient presenting with significant weight loss, fatigue, and unexplained anaemia, especially in those with a long history of smoking. Recognition of paraneoplastic syndrome could lead to an early cancer diagnosis, that may significantly improve long term outcome for the patient.

Notable features of this case include clinical presentation with symmetric polyarthritis, mimicking rheumatoid arthritis, and the discovery of synchronous malignancies – adenosquamous lung carcinoma and in situ colorectal carcinoma.
